# Amphiregulin can predict treatment resistance to palliative first-line cetuximab plus FOLFIRI chemotherapy in patients with *RAS* wild-type metastatic colorectal cancer

**DOI:** 10.1038/s41598-021-03197-9

**Published:** 2021-12-10

**Authors:** Sang-A Kim, Hyejoo Park, Kui-Jin Kim, Ji-Won Kim, Ji Hea Sung, Milang Nam, Ju Hyun Lee, Eun Hee Jung, Koung Jin Suh, Ji Yun Lee, Se Hyun Kim, Jeong-Ok Lee, Jin Won Kim, Yu Jung Kim, Jee Hyun Kim, Soo-Mee Bang, Jong Seok Lee, Keun-Wook Lee

**Affiliations:** 1 Division of Hematology and Medical Oncology, Department of Internal Medicine, Seoul National University Bundang Hospital, Seoul National University College of Medicine, Seongnam, 13620 Republic of Korea; 2 Biomedical Research Institute, Seoul National University Bundang Hospital, Seongnam, 13620 Republic of Korea

**Keywords:** Colon cancer, Colon cancer, Outcomes research

## Abstract

Amphiregulin (AREG) is an epidermal growth factor receptor (EGFR) ligand. The aim of this study was to investigate the effects of baseline plasma AREG levels in *KRAS*, *NRAS*, and *BRAF* wild-type metastatic colorectal cancer (CRC) on treatment outcome with palliative first-line cetuximab + FOLFIRI chemotherapy. Chemotherapy outcomes were analyzed based on baseline plasma AREG levels. The clinical findings were further validated using an in vitro model of CRC. Among 35 patients, the progression-free survival (PFS) was significantly inferior in patients with high AREG than in those with low AREG levels: 10.9 *vs*. 24.2 months, respectively (*p* = 0.008). However, after failure of first-line chemotherapy, AREG levels were associated with neither PFS (4.8 *vs.* 11.6 months; *p* = 0.215) nor overall survival (8.4 *vs.* 13.3 months; *p* = 0.975). In SNU-C4 and Caco-2 cells which were relatively sensitive to cetuximab among the seven CRC cell lines tested, AREG significantly decreased the anti-proliferative effect of cetuximab (*p* < 0.05) via AKT and ERK activation. However, after acquiring cetuximab resistance with gradual exposure for more than 6 months, AREG neither increased colony formation nor activated AKT and ERK after cetuximab treatment. Our results suggest that plasma AREG is a potential biomarker to predict clinical outcomes after cetuximab-based chemotherapy.

## Introduction

Palliative first-line chemotherapy with anti-EGFR (epidermal growth factor receptor) monoclonal antibody, cetuximab, or panitumumab, combined with conventional cytotoxic chemotherapy, has shown preferable outcomes in phase III trials^1–4^. Of note, the antitumor effect was conspicuous in the patients with extended *RAS* wild-type cancers^5^. However, although overall survival (OS) reached over 2 years with this treatment strategy, most of the patients are deemed to encounter treatment resistance. *KRAS*, *NRAS*, and *BRAF* (*RAS/BRAF*) mutation status is currently the only factor that predicts the response to cetuximab treatment. However, in clinical practice, the treatment response is varied even among *RAS/BRAF* wild-type patients.

Amphiregulin (AREG) is an EGFR ligand, which is associated with normal tissue development and proliferation and with a wide variety of human cancers including colorectal cancer^6–9^. In colorectal cancer, AREG is known to act competitively with anti-EGFR monoclonal antibodies and activate downstream signaling, and thus may be involved with cetuximab resistance^10^. It has been speculated that patients with high AREG levels may have worse treatment outcomes with anti-EGFR therapy. However, in some studies, conflicting results have been reported on the effects of AREG on anti-EGFR treatment outcomes^11–13^.

Therefore, in this study, we aimed to investigate the effects of baseline plasma AREG levels in patients with colorectal cancer treated with palliative first-line cetuximab plus FOLFIRI regimen and subsequent second-line chemotherapy. In addition, we conducted an in vitro study to further define the molecular mechanisms of AREG in cetuximab-na**ï**ve and cetuximab-resistant colorectal cancer cell lines.

## Results

### High baseline plasma AREG levels are associated with inferior progression-free survival

From May 2015 to September 2019, a total of 35 patients were consecutively included in this study. All patients were treated with palliative first-line chemotherapy consisting of cetuximab plus FOLFIRI. Among them, 21 patients (60.0%) had left-sided colorectal cancer and most of the cancers (n = 29, 82.9%) showed moderate differentiation. All patients were evaluated for *RAS* mutation; no one had *RAS* mutation at the time of diagnosis. *BRAF* mutation was evaluated in 34 patients; 2 patients (5.9%) had *BRAF* mutant cancer (Table 1).

**Table 1:** Baseline characteristics of patients.

	Patients (n = 35)
Age at diagnosis, year (range)	66 (24–85)
**Sex, n (%)**	
Male	20 (57.1)
Female	15 (42.9)
**ECOG PS, n (%)**	
0–1	33 (94.3)
2	2 (5.7%)
**Primary site, n (%)**	
Right-sided	5 (14.3%)
Left-sided	21 (60.0%)
Rectum	9 (25.7%)
**Differentiation, n (%)**	
Well differentiated	2 (5.7%)
Adenocarcinoma, moderately differentiated	29 (82.9%)
Adenocarcinoma, poorly differentiated	3 (8.6%)
Adenocarcinoma, NOS	1 (2.9%)
*KRAS* mutation, n (%)	0 (0.0%)
*NRAS* mutation, n (%)	0 (0.0%)
*BRAF* mutation, n (%)^a^	2 (5.9%)
**MSI, n (%)**^**a**^	
MSS	30 (85.7%)
MSI-L	4 (11.4%)
MSI-H	1 (2.9%)
CEA (ng/mL)	23.1 (1.4–15,480.0)
CA19-9 (U/mL)	72.5 (2.0–16,900.0)

*ECOG PS* Eastern Cooperative Oncology Group performance status, *NOS* not otherwise specified, *MSI* microsatellite instability, *MSS* microsatellite stable, *MSI-L* microsatellite instability-low, *MSI-H* microsatellite instability-high.

^a^Percentage of *RAS*, *BRAF* mutation, and MSI status was calculated for patients whose data were available.

**Figure 1:**
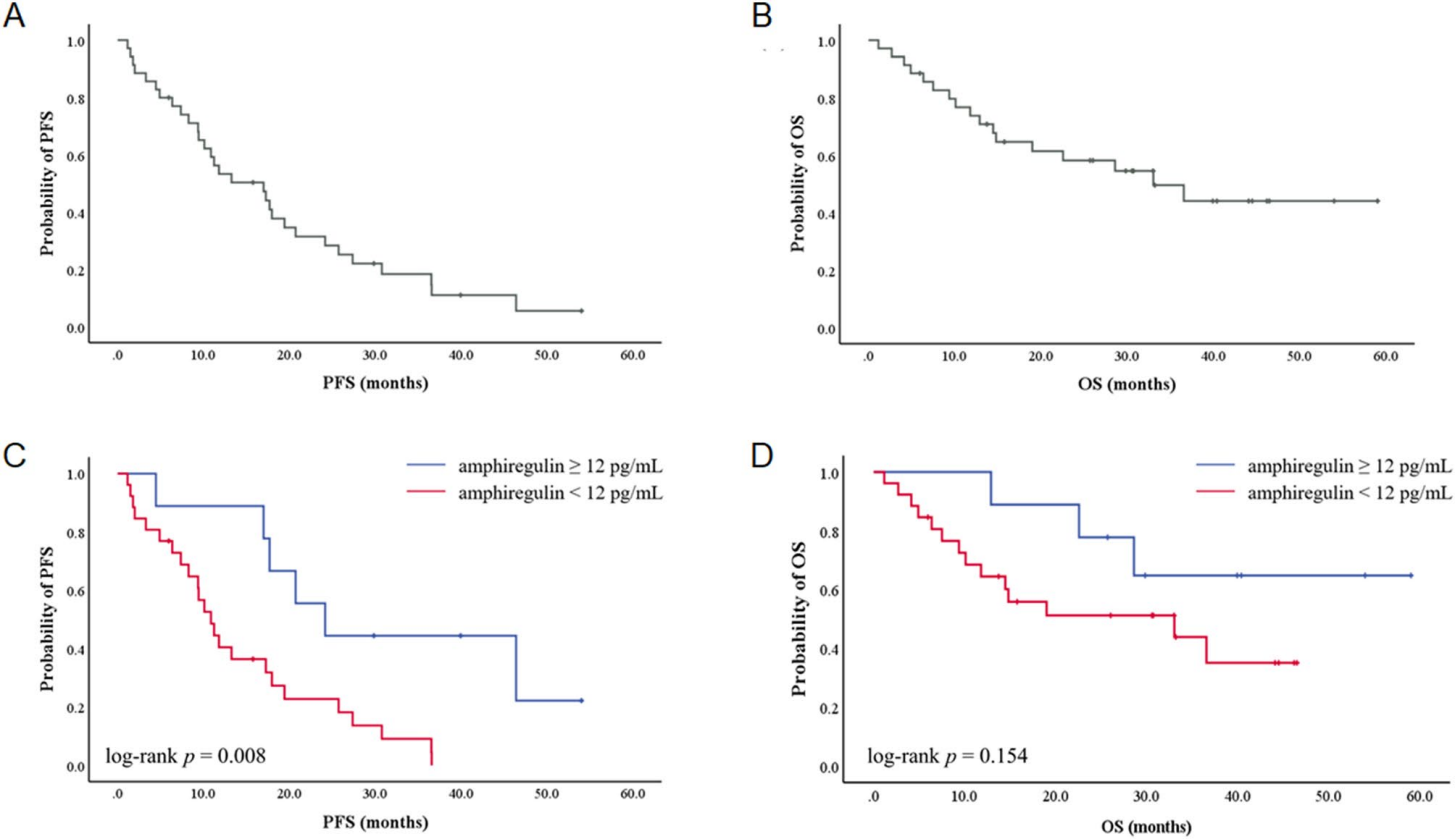
Kaplan–Meier survival analysis of patients who were treated with palliative first-line cetuximab + FOLFIRI chemotherapy. (**A**) Progression-free survival (PFS), (**B**) overall survival (OS) of the entire patient population, (**C**) PFS, (**D**) OS according to plasma AREG levels.

The median follow-up time was 19.1 months (range 1.2–56.0 months). Among patients whose disease had target lesions defined by RECIST version 1.1 (n = 34), 21 patients (60.0%) showed partial response (Table 2). In the entire patient cohort, the median PFS and OS were 17.0 months (95% CI 15.0–51.1 months) and 33.0 months (95% confidence interval [CI] 8.7–25.3 months), respectively (Fig. 1A,B).

**Table 2:** Best objective response after chemotherapy.

	No. of patients (%)
Partial response	21 (60.0)
Stable disease	10 (28.6)
Progressive disease	3 (8.6)
Not evaluable*	1 (2.9)
Total	35 (100)

*One patient was lost to follow-up after the first cycle of chemotherapy.

The median plasma AREG level was 33.6 pg/mL (range 0–1570.54 pg/mL). Using a maximal chi-square test, the cut-off value for AREG level was defined as 12.00 pg/mL. Comparing patients with high (n = 26) vs low plasma AREG levels, PFS was significantly inferior (10.9 months [95% CI 8.0–13.8 months] *vs*. 24.2 months [95% CI 14.1–34.2]; *p* = 0.008, respectively) (Fig. 1C). Patients with high plasma AREG levels also showed a trend toward inferior OS (33.0 months [95% CI 0.0–66.1 months] vs. not reached, respectively; *p* = 0.154) (Fig. 1D).

### Baseline plasma AREG levels are correlated with neither PFS nor OS after progression of palliative first-line chemotherapy

**Figure 2:**
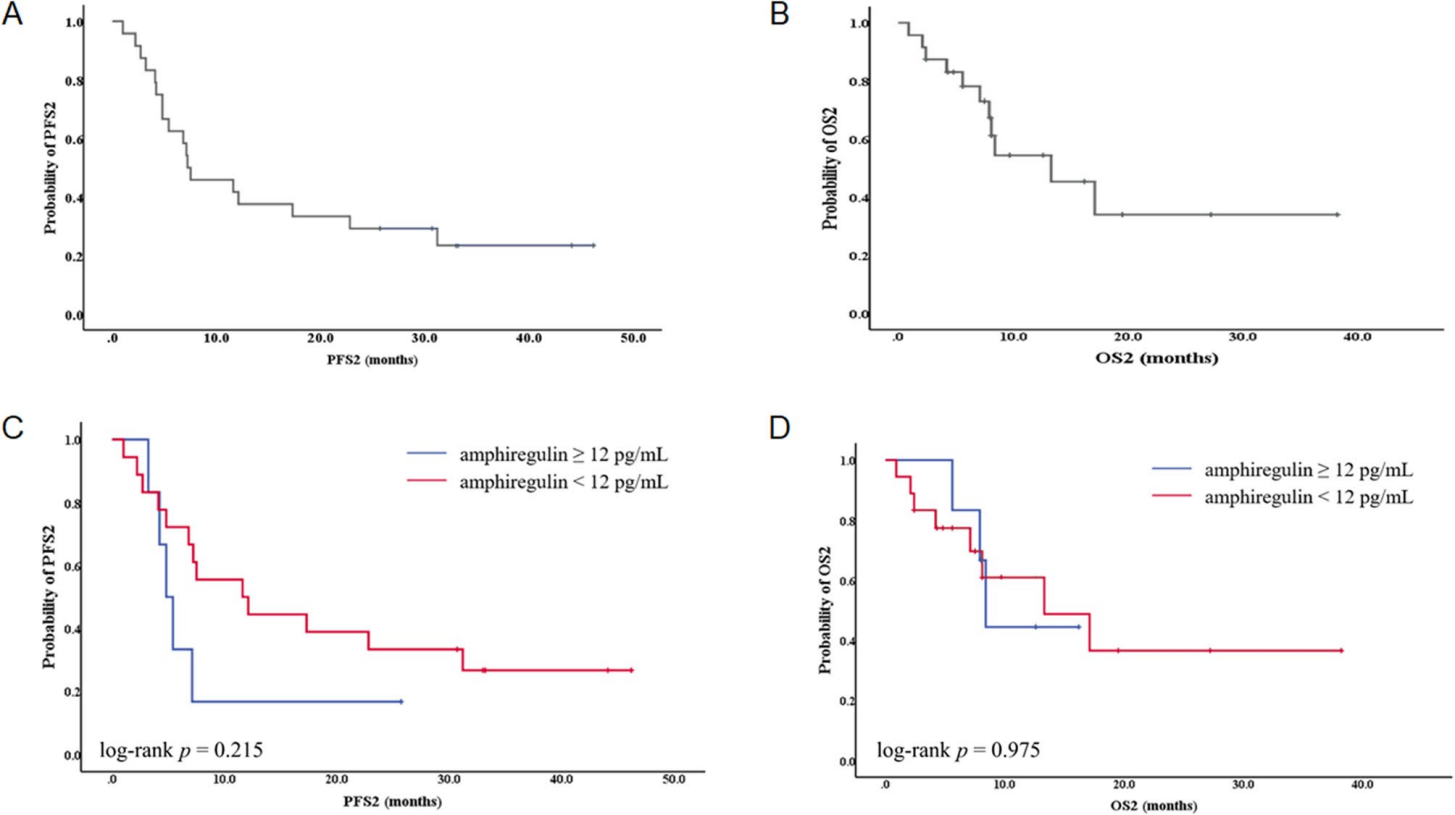
Kaplan–Meier survival analysis of patients who were treated with palliative 2nd-line chemotherapy after progression of 1st-line cetuximab + FOLFIRI treatment. (**A**) Progression-free survival (PFS), (**B**) overall survival (OS) among the patients who showed progression with first-line palliative chemotherapy and (**C**) PFS, (**D**) OS according to plasma AREG levels.

PFS2 and OS2 were evaluated among the patients who showed disease progression with cetuximab + FOLFIRI (n = 24). Most of the patients (n = 20) were treated with bevacizumab + FOLFOX after progression, while the others (n = 3) received cetuximab + FOLFIRI retreatment (n = 1), regorafenib (n = 1), or radiation (n = 1). One patient did not receive any subsequent treatment. The median PFS2 and OS2 were 7.2 months (95% CI 1.5–12.9 months) and 13.3 months (95% CI 5.0–21.6 months), respectively (Fig. 2A,B). Baseline plasma AREG levels were associated with neither PFS2 (4.8 months [95% CI 3.4–6.3 months] *vs*. 11.6 months [95% CI 2.1–21.1 months]; *p* = 0.215) (Fig. 2C) nor OS2 (8.4 months [95% CI 7.3–9.5 months] *vs*. 13.3 months [95% CI 2.1–24.5 months]; *p* = 0.975) (Fig. 2D).

### AREG decreases cellular response to cetuximab

**Figure 3:**
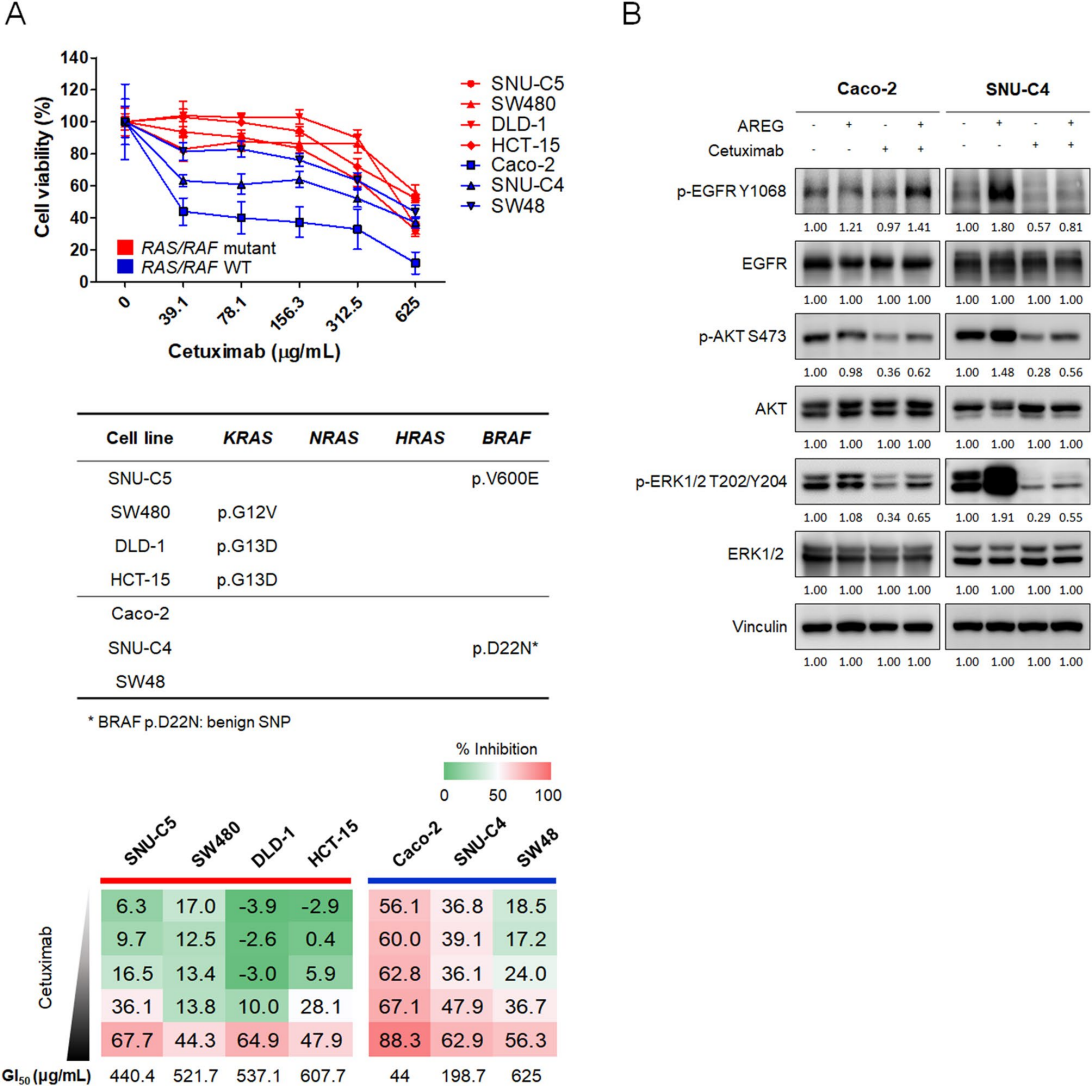
Effect of AREG on cetuximab-induced anti-proliferative effects and EGFR signaling pathways in colorectal cancer cells. (**A**) Cetuximab cytotoxicity assay in various colorectal cancer cell lines. Serially diluted cetuximab was added for 5 days and cell viability was measured using CellTiter-Glo. (**B**) western blot of EGFR signaling molecules after treatment with cetuximab and 50 ng/mL AREG. Cetuximab was added at GI_50_ concentration for 15 min. AREG was added to SNU-C4 and Caco-2 cells 15 min and 24 h prior to cetuximab treatment, respectively. Serum starvation for 24 h was conducted before the addition of cetuximab and AREG.

The anti-proliferative effects of cetuximab were evaluated in seven human colorectal cancer cells, SNU-C5, SW480, DLD-1, HCT-15, Caco-2, SNU-C4, and SW48 (Fig. 3A). Cell lines with *KRAS* or *BRAF* hotspot mutations were resistant to cetuximab (GI_50_ ≥ 440.4 μg/mL), while, among those without *KRAS* and *BRAF* hotspot mutations, Caco-2 and SNU-C4 were relatively sensitive to cetuximab with GI_50_ values of 44.0 μg/mL and 198.7 μg/mL, respectively. Based on cetuximab sensitivity, Caco-2 and SNU-C4 cell lines were selected for further study.

To determine the effect of cetuximab combined with AREG on the EGFR signaling pathways, Western blot analyses were conducted (Fig. 3B). AREG increased the phosphorylation of EGFR Y1068 (p-EGFR), which is the most sensitive site to EGFR ligand stimulation among p-EGFR residues, in Caco-2 and SNU-C4 cells^14,15^. In AREG-treated Caco-2 cells, the changes of EGFR downstream molecules involving phosphorylated AKT (p-AKT) and phosphorylated ERK1/2 (p-ERK1/2) were not apparent. In contrast, the phosphorylation of AKT and ERK1/2 strongly increased by adding AREG to SNU-C4 cells, indicating a more responsive phenotype against AREG in terms of signaling pathway activation compared with Caco-2 cells. Further, cetuximab monotherapy decreased the phosphorylation of EGFR, AKT, and ERK1/2 in both cell lines. To examine the effects of AREG on cetuximab response, AREG was administered prior to cetuximab treatment in Caco-2 and SNU-C4 cells. In both Caco-2 and SNU-C4 cells, AREG partially restored the cetuximab-induced inhibition of EGFR downstream molecules, AKT and ERK1/2.

**Figure 4:**
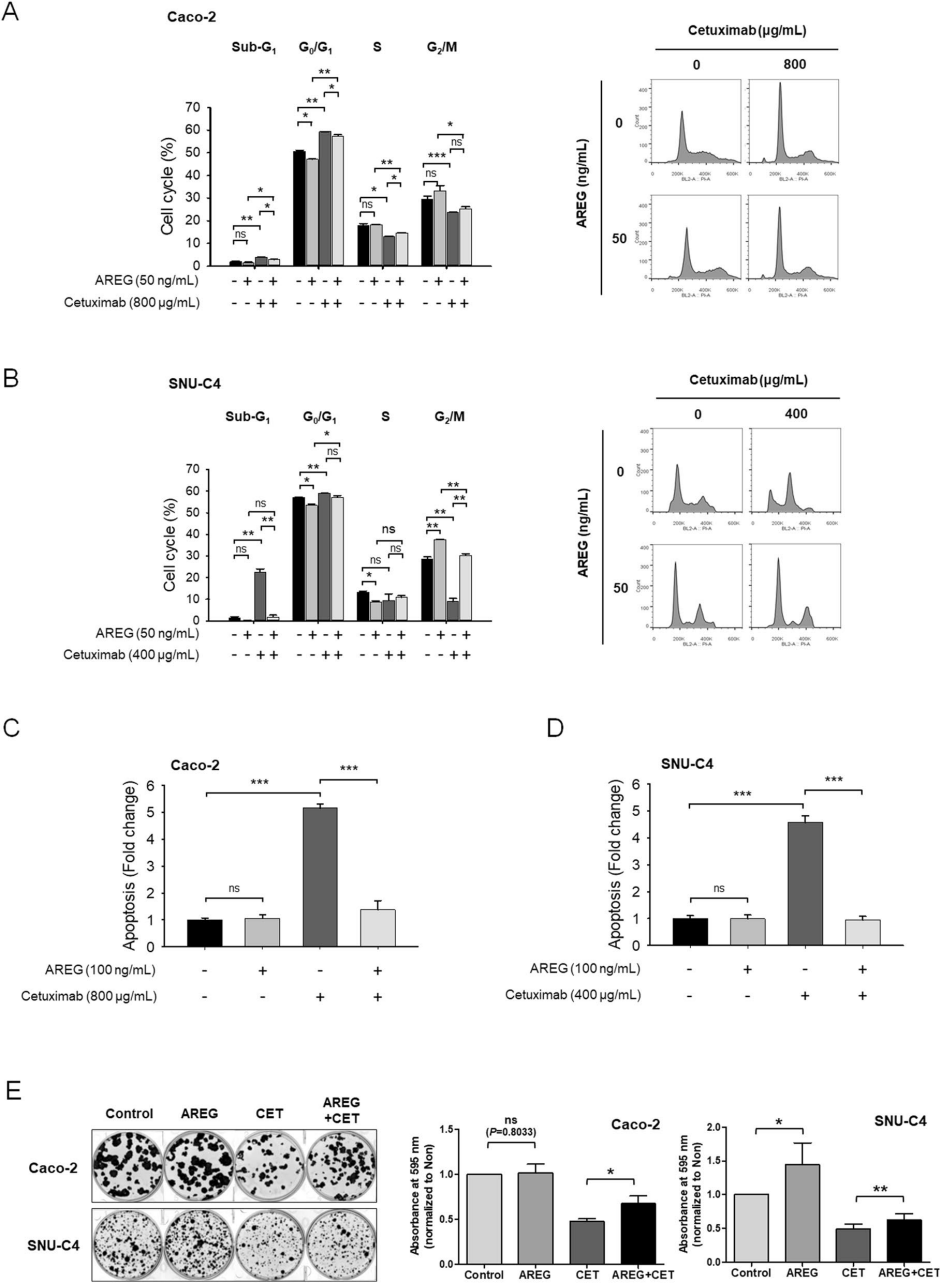
Effect of AREG on cetuximab-mediated cell cycle, apoptosis, and colony forming activities in colorectal cancer cells. In (**A**) Caco-2 and (**B**) SNU-C4, cell cycle analysis was conducted using flow cytometry after propidium iodide (PI) staining. The cells were seeded in 60-mm plates, and then treated with or without cetuximab or AREG for 72 h. (**C**) Caco-2 and (**D**) SNU-C4 cells were treated with the indicated concentrations of cetuximab. The cells were also exposed to the indicated concentrations of AREG for 96 h. Apoptosis was measured using the Caspase-Glo^®^ 3/7 assay kit. Results indicate mean ± standard deviation. The values were compared using the Student’s t-test. *p < 0.05; **p < 0.01; ***p < 0.001. (**E**) Colony-forming assays were conducted to evaluate the long-term effect of AREG. AREG 50 ng/mL and cetuximab (10 μg/mL for Caco-2 and 20 μg/mL for SNU-C4) were added to the cells for 3 to 4 weeks. The colonies were quantified for optical density at 595 nm.

To assess the effect of cetuximab in the presence or absence of AREG on the cell cycle in Caco-2 and SNU-C4 cells, we conducted flow cytometry (Fig. 4A,B). We sought that cetuximab monotherapy significantly increased the G0/G1 phase but decreased the S and G2/M phase in Caco-2 and SNU-C4 cells. However, in the presence of AREG, cetuximab-mediated cell cycle alteration was partially reversed in Caco-2 and SNU-C4 cells. Particularly, in the presence of AREG, subG1 phase was significantly repressed with the cetuximab treatment in both cells, indicating that AREG might reduce the activity of cetuximab in Caco-2 and SNU-C4 cells.

To verify whether AREG disturbs cetuximab-mediated apoptosis, the caspase-3/7-positive apoptotic cells were measured in Caco-2 and SNU-C4 cells with AREG in the presence or absence of cetuximab (Fig. 4C,D). AREG alone did not alter the number of apoptotic rates. Notably, in the presence of AREG, the cetuximab-mediated apoptotic cells were significantly suppressed in both Caco-2 and SNU-C4 cells compared with AREG-free condition.

In addition, the colony-forming assays were carried out to analyze the prolonged effect of cetuximab in the presence or absence of AREG on the proliferation of Caco-2 and SNU-C4 cells (Fig. 4E). We observed that cetuximab monotherapy significantly decreased the growth of colonies compared with the control in Caco-2 and SNU-C4 cells. In the absence of cetuximab treatment, high AREG did not significantly increase cell proliferation in Caco-2 cells, whereas in SNU-C4 cells, the proliferation was significantly increased in the presence of AREG relative to that in the control. The colony-forming assay results appeared to be correlated with the western blot data (Fig. 3B): SNU-C4 cells showed higher susceptibility to the effect of AREG on EGFR downstream molecules, AKT and ERK1/2, possibly resulting in higher proliferation, compared with Caco-2 cells. Notably, in the presence of AREG, the anti-proliferative effects of cetuximab were significantly decreased in both Caco-2 and SNU-C4 cells compared to AREG-free environment.

### AREG does not confer additional cetuximab resistance in cetuximab acquired resistance cell lines

**Figure 5:**
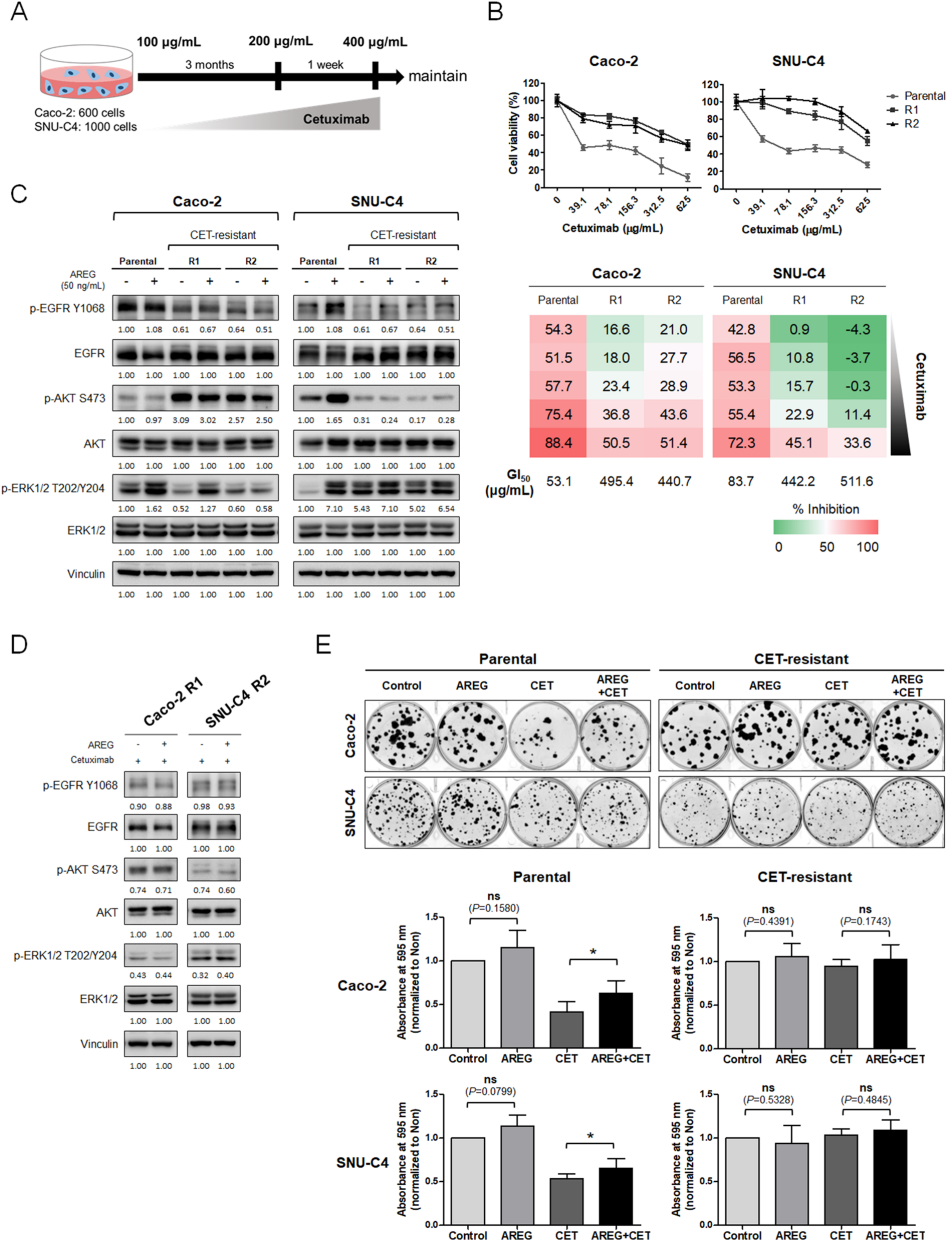
Effect of AREG on cell proliferation and EGFR signaling pathways in cetuximab-resistant colorectal cancer cells. (**A**) Scheme of the establishment of the cetuximab acquired resistance model (CET-R). Cetuximab was treated in a dose-escalating manner for 3 months. After acquiring resistance, cells were maintained with 400 μg/mL cetuximab. (**B**) Cytotoxicity assay was conducted to evaluate the resistance to cetuximab between parental and CET-R. Cetuximab was administered for 5 days, and cell viability was confirmed using CellTiter-Glo. (**C**) Western blotting of signaling molecules for an examination of the response to AREG in CET-R. 50 ng/mL AREG was added 15 min after 24 h serum starvation. Screening of EGFR down signaling molecules by Western blotting (**D**) and colony-forming assay (**E**) were carried out to check effects of AREG on cetuximab in CET-R. The conditions of treatment with cetuximab and AREG were equal in Figs. 3B and 4E.

We established cetuximab-resistant cell lines (Caco-2_R and SNU-C4_R) and their corresponding parental cells (Caco-2_P and SNU-C4_P) to evaluate the effects of AREG in cetuximab-resistant conditions (Fig. 5A). Cetuximab resistance was acquired by gradual dose escalation for 3 months. Caco-2_R and SNU-C4_R cells represented approximately eightfold (R1: 495.4 and R2: 440.7 μg/mL) and fivefold (R1: 442.2 and R2: 511.6 μg/mL) higher GI_50_ values to cetuximab, respectively, compared with their corresponding parental cells (Fig. 5B).

We examined the influence of AREG on the EGFR signaling pathways by western blot analysis in Caco-2_R and SNU-C4_R cells (Fig. 5C). In line with our western blot data in the previous section (Fig. 3B), AREG increased the phosphorylation of EGFR, AKT, and ERK1/2 in SNU-C4_P cells, whereas SNU-C4_R cells did not respond to AREG. In contrast, in Caco-2_P cells, high AREG did not cause AKT phosphorylation, but led to ERK1/2 phosphorylation. High AREG slightly increased the expression of p-ERK1/2 in Caco-2_R1 cells but had no such effect in Caco-2_R2. In Caco-2_R1 and Caco-2_R2 cells, p-AKT slightly decreased in the presence of AREG (Supplementary Fig. S1).

Further, in cetuximab resistant cells, EGFR and its downstream signaling molecules were examined after cetuximab treatment in the presence or absence of AREG (Fig. 5D). Based on the anti-proliferation assay data (Fig. 5B), relatively more resistant clones, Caco-2_R1 and SNU-C4_R2, were selected for the study. In these cetuximab-resistant cells, additional western blot analysis showed that, after cetuximab treatment, the presence or absence of AREG did not elicit apparent differences in terms of EGFR downstream signaling (Fig. 5D).

Next, we carried out colony-forming assays to determine the prolonged effect of AREG on proliferation in cetuximab-resistant cells (Fig. 5E). Colony-forming assays of Caco-2_P and SNU-C4_P cells showed similar results as in cetuximab-naïve cells (Fig. 4E). However, in Caco-2_R1 and SNU-C4_R2 cells, the anti-proliferative effects of cetuximab were not significant both in the presence and absence of AREG.

## Discussion

This study was conducted to investigate the effects of AREG on cetuximab response in patients with metastatic colorectal cancer and determine the mode of action via the EGFR signaling pathway using in vitro experiments with both cetuximab-naïve and resistant colorectal cancer cell lines. Our results indicated that high baseline plasma AREG levels could predict PFS after first-line cetuximab plus FOLFIRI chemotherapy in patients with *RAS/BRAF* wild-type colorectal cancer. Our in vitro study also indicated that AREG significantly enhanced the growth of cetuximab-sensitive *RAS/BRAF* wild-type colorectal cancer cell lines with increased phosphorylation of AKT and ERK1/2. However, after progression of first-line chemotherapy, the plasma AREG levels could not predict treatment outcomes of second-line non-EGFR-directed treatment, suggesting that AREG may not be a prognostic biomarker in these patients. In line with this clinical finding, in cetuximab-resistant cell lines, AREG neither impeded the signal transduction inhibition by cetuximab nor was associated with the cetuximab-induced anti-proliferative effects.

It is still controversial whether AREG protein or mRNA expression levels are prognostic factors in patients with colorectal cancer who did not undergo cetuximab- or panitumumab-based chemotherapy. For example, Ohchi et al. showed that higher AREG expression by IHC was significantly associated with worse relapse-free survival and OS^16^. In contrast, Stahler et al. reported that AREG mRNA expression in tumor tissue was not prognostic^17^. Besides, several studies demonstrated that higher AREG expression levels by tissue immunohistochemistry (IHC) and serum ELISA were associated with worse pathologic parameters^18–20^.

Other studies evaluated the predictive role of mRNA expression of AREG in the patients receiving cetuximab- or panitumumab-based treatment. In most studies, mRNA expression of AREG predicted better response to anti-EGFR treatment. However, mRNA-based analyses are less likely to be reproducible and thus difficult to adopt in clinical practice. Yonesaka et al. evaluated plasma AREG levels by ELISA in colorectal cancer patients treated with cetuximab in combination with cytotoxic chemotherapy^21^. However, treatment in the patients were heterogeneous in terms of combined cytotoxic chemotherapy and prior lines of treatment. In addition, this study evaluated the role of plasma AREG levels in combination with heregulin, therefore, further validation is required to determine whether plasma AREG levels by ELISA could be a predictive biomarker for cetuximab-based treatment.

Furthermore, our study group previously conducted a biomarker study of serum AREG in patients with HER2-positive breast cancer treated with trastuzumab plus taxane and found that high AREG levels were associated with poor clinical outcomes^22^. EGFR and HER2 belong to the epidermal growth factor family of receptor tyrosine kinases (ErbBs), which includes EGFR, HER2, HER3, and HER4^23^. HER2 is a receptor which has no identified ligand but shares the same downstream pathway with EGFR. AREG is a ligand of EGFR, which can form a heterodimer with HER2, leading to down-stream signaling activation^24^. Therefore, our interpretation in our previous study was that AREG indirectly confers trastuzumab resistance via EGFR activation in HER2-positive breast cancer. In contrast, in the present study, AREG-induced cetuximab resistance may be attributed to direct EGFR activation in *RAS/BRAF* wild-type colorectal cancer.

We further analyzed the biological mechanisms of AREG using in vitro experiments. In our clinical data, plasma AREG was a negative predictive biomarker of cetuximab-based treatment, which was validated in our in vitro study as well. However, it was no longer predictive after patients acquired cetuximab resistance. Thus, it is speculated that AREG interferes with the inhibitory effect of cetuximab on EGFR. To prove the hypothesis, we generated cells with acquired cetuximab resistance using cetuximab-sensitive Caco-2 and SNU-C4 cell lines. As a result, AREG could not activate the EGFR down-stream signaling in cetuximab-resistant Caco-2 and SNU-C4 cells.

Based on these findings, it is hypothesized that AREG inhibition might have a therapeutic potential in patients receiving anti-EGFR treatment. Several preclinical studies have investigated the effect of AREG inhibition using anti-AREG antibodies or AREG shRNA in various human cancer cell lines^25–27^. However, there is still no clinical data on AREG inhibition in patients with high plasma AREG levels receiving anti-EGFR treatment. Our results may provide evidence of an AREG-targeting strategy in patients with *RAS/BRAF* wild-type colorectal cancer receiving cetuximab-based chemotherapy.

One of the limitations of our study is a small patient population and lack of validation cohort. Hence, it should be further validated in a larger patient cohort whether amphiregulin is an independent biomarker in this patient population. In addition, our experiment did not include various mechanisms of action of cetuximab including the antibody-dependent cell mediated cytotoxicity^28^. In this regard, further research is warranted.

In conclusion, high baseline plasma AREG levels predicted worse PFS in patients with *RAS*/*BRAF* wild-type metastatic colorectal cancer treated with palliative first-line cetuximab plus FOLFIRI chemotherapy, but not with non-EGFR-directed second-line treatment. AREG reversed the inhibitory effects of cetuximab in *RAS*/*BRAF* wild-type cetuximab-naïve colorectal cancer cells, but not in cetuximab-resistance cells in vitro. Our results suggest that plasma AREG is a potential biomarker to predict clinical outcomes after cetuximab-based chemotherapy.

## Patients and methods

### Patient population

In total, 35 consecutive patients were enrolled in this study between May 2015 and September 2019. The patients were treated with palliative first-line cetuximab plus FOLFIRI chemotherapy at Seoul National University Bundang Hospital, Seongnam, Korea. The chemotherapy regimen consisted of intravenous cetuximab 500 mg/m^2^ combined with FOLFIRI. The FOLFIRI regimen consisted of an intravenous infusion of irinotecan 180 mg/m^2^ on day 1, followed by leucovorin 400 mg/m^2^ infusion and 5-fluorouracil 400 mg/m^2^ bolus, and then 5-fluorouracil 2400 mg/m^2^ infusion over 46 h every 2 weeks. Before the first dose of cetuximab + FOLFIRI treatment, blood samples were collected after obtaining informed consent during two preceding prospective studies: pharmacogenomic study in patients with advanced solid tumors receiving palliative chemotherapy (IRB registration No. B-1603/340-305) and biomarker study in patients with metastatic colorectal cancer (IRB registration No. B-1211/180-007). The present study was conducted using these consecutively collected blood samples. Retrospective medical record reviews were conducted for these patients. The study was approved by the institutional review board of Seoul National University Bundang Hospital (IRB registration No. B-2102-667-301) and performed in accordance with the ethical principles of the Declaration of Helsinki.

### Plasma sample collection and AREG quantification

Plasma was separated from the peripheral blood samples of patients by centrifugation at 3000×*g* for 10 min. Human recombinant AREG levels in the plasma samples were quantified using a Quantikine Human AREG ELISA (enzyme-linked immunosorbent assay) kit (DAR00, R&D Systems, Minneapolis, MN, USA) according to the manufacturer’s protocol.

### Cell culture

The human colorectal cancer cell lines, SNU-C5, SW480, DLD-1, HCT-15, Caco-2, and SNU-C4, were obtained from the Korean Cell Line Bank (KCLB, Seoul, Korea). The cells were cultured with RPMI 1640 medium (LM011-51, WELGENE, Gyeongsan, Korea) supplemented with 10% fetal bovine serum (FBS) (26140079, Gibco, Waltham, MA, USA) and 1% penicillin–streptomycin (LS202-02, WELGENE) solution. SW48 was purchased from the American Type Culture Collection (ATCC, Manassas, VA, USA) and cultured with DMEM medium (11995065, Gibco) supplemented with 10% FBS and 1% penicillin–streptomycin. Cetuximab-resistant cells were maintained in a medium with 400 μg/mL cetuximab. All the cells were maintained every 3–4 days and cultured at 37 ℃ in a humidified 5% CO_2_ incubator.

### Cell viability assay

Cells were seeded in 384-well plates (781080, Greiner Bio-One, Kremsmünster, Austria) at 1,000 cells/well 1 day prior to cetuximab treatment. At day 0 and 5, cell viability was measured by CellTiter-Glo luminescent cell viability assay (G7573, Promega, Madison, WI, USA) according to the manufacturer’s protocol. Luminescence was measured with the Synergy H1 microplate reader (BioTek, Winooski, VT, USA). The half-maximal growth inhibition (GI_50_) values were determined using CalcuSyn 2.0 software (Biosoft, Cambridge, UK).

### Establishment of acquired cetuximab-resistant colorectal cancer cell lines

Cetuximab-resistant Caco-2 and SNU-C4 cell lines were generated by continuous exposure to increasing concentrations of cetuximab up to 400 μg/mL for 3 months, and then maintained in cetuximab 400 μg/mL-containing media for more than 6 months (Fig. 3A). Parental Caco-2 or SNU-C4 cells were simultaneously maintained by continuous exposure to cetuximab-free media. Parental (P) or cetuximab resistant (R) cells were designated as Caco-2_P, Caco-2_R (clone 1 and 2), SNU-C4_P, and SNU-C4_R (clone 1 and 2). Caco-2_R (clone 1 and 2) and SNU-C4_R (clone 1 and 2) cell lines showed approximately five to ninefold higher GI_50_ values than their corresponding parental cells.

### Western blot analysis

The cells were washed with cold Dulbecco’s phosphate-buffered saline (PBS) (LB 001-02, WELGENE) and then lysed using radio immunoprecipitation assay (RIPA) buffer with protease and phosphatase inhibitors. The bicinchoninic acid (BCA) protein assay kit (23227, Thermo Fisher Scientific, Waltham, MA, USA) was used for protein quantification. Protein samples were separated on 8–10% sodium dodecyl sulfate (SDS)-polyacrylamide gel and then transferred onto 0.45 μM pore polyvinylidene fluoride (PVDF) membrane (IPVH00010, Millipore, Burlington, MA, USA). The membrane was blocked with 5% skim milk (70166, Sigma-Aldrich, St. Louis, MO, USA) solution and was probed with primary antibody at 4 ℃ overnight. After incubation with horseradish peroxidase (HRP)-conjugated secondary antibody for 2 h at room temperature, the blot was detected using ChemiDoc Touch Imaging System (BioRad, Hercules, CA, USA). Protein expression was analyzed by ImageJ software (NIH, Baltimore, MD, USA).

### Antibodies and reagents

Phospho-EGFR (Y1068) (#3777), EGFR (#2646), phospho-AKT (S473) (#4058), AKT (#4685), phospho-ERK1/2 (T202/Y204) (#4376), ERK1/2 (#4695), and vinculin (#13901) primary antibodies were purchased from Cell Signaling Technology (Danvers, MA, USA). Vinculin was used as loading control. HRP-conjugated anti-rabbit (#111-035-003) and mouse (#115-035-003) secondary antibodies were obtained from Jackson ImmunoResearch (West Grove, PA, US). Cetuximab (A2000) was purchased from Selleckchem (Houston, TX, USA). The recombinant human AREG (262-AR-100) was purchased from R&D Systems.

### Cell cycle analysis

The Caco-2 and SNU-C4 cells were plated in 60-mm culture dishes at a density of 1 × 10^6^ cells per dish for 72 h, and then treated with AREG and/or cetuximab. Next, the cells were harvested at 72 h by trypsinization, centrifugation, and fixation with 70% ethanol. A staining solution containing 0.05 μg/mL of propidium iodide (PI) and 0.2 mg/mL of RNase was used for DNA staining. Attune^®^ NxT cytometer (Thermo Fisher Scientific) was used for cell cycle analysis. The flow cytometry data was processed using FlowJo software (BD Biosciences, San Jose, CA, USA).

### Apoptosis assay

Apoptosis assays were performed according to manufacturer’s recommendations using the Caspase-Glo^®^ 3/7 assay kit (Promega). Significant differences between values obtained in DMSO control groups and different treatment groups were determined using the Student’s t-test.

### Colony-forming assay

Cells were seeded in 6-well plates at 1000 cells/well (30006, SPL Life Science, Pocheon, Korea). The treatments, 10 and 20 μg/mL of cetuximab and 50 ng/mL of AREG, were added three times weekly for 3 or 4 weeks to SNU-C4 or Caco-2, respectively. The cell colonies were stained with Coomassie brilliant blue R-250 solution (1610436, BioRad) for 2 h at room temperature. The representative colony images were photographed by ChemiDoc Touch Imaging System. The procedure of colony de-staining was performed using a 1% SDS solution to analyze the relative colony area. The relative colony area was determined at 595 nm using a Synergy H1 microplate reader. Protein expression was analyzed by ImageJ software (NIH, Baltimore, MD, USA).

### Statistical analysis

The Eastern Cooperative Oncology Group (ECOG) scale was used to evaluate performance status (PS) of patients. *KRAS*, *NRAS*, and *BRAF* mutation status was evaluated using polymerase chain reaction (PCR) or next generation sequencing as a part of clinical practice. Microsatellite instability was determined according to the revised Bethesda guidelines (14). Disease status was evaluated using computed tomography (CT) scans or magnetic resonance imaging (MRI). Treatment response was assessed in accordance with the response evaluation criteria in solid tumor (RECIST) version 1.1^29^.

To identify the optimal cut-off value for plasma AREG levels, Maxstat R package (a maximal chi-square method) was used in R 4.0.3 (R Development Core Team, Vienna, Austria). PFS was defined from the start of systemic chemotherapy to the date of documented disease progression, relapse, or death of any cause. The second PFS (PFS2) was defined from the start of second-line anticancer treatment to the date of documented disease progression, relapse, or death of any cause. OS was measured from the start day of systemic chemotherapy to the date of death of any cause. The second OS (OS2) was defined from the start of the second-line anticancer treatment to the date of death of any cause. The survival outcomes were calculated using the Kaplan–Meier method and compared with the log-rank test.

All in vitro experiments were conducted at least three times independently. The differences between groups were evaluated by Student’s *t*-test using GraphPad Prism 5 software (San Diego, CA, USA). Error bars represent mean ± standard deviation. Two-sided *p*-values < 0.05 were considered statistically significant (*p < 0.05, **p < 0.01, and ***p < 0.001).

## Supplementary Information

Supplementary Figures.

## Data Availability

The datasets generated during and/or analysis during the current study are available from the corresponding author on reasonable request.
