# Supplementary material for: Amphiregulin can predict treatment resistance to palliative first-line cetuximab plus FOLFIRI chemotherapy in patients with RAS wild-type metastatic colorectal cancer
Source: Sci Rep. 2021 Dec 10;11:23803. doi: 10.1038/s41598-021-03197-9 (PMC8664812; doi:10.1038/s41598-021-03197-9)
Supplement: Supplementary file 1 — Supplementary Figures. [file 41598_2021_3197_MOESM1_ESM.docx]

**Amphiregulin can predict treatment resistance to palliative first-line cetuximab plus FOLFIRI chemotherapy in patients with *RAS* wild-type metastatic colorectal cancer**

**Running title:** Amphiregulin in RAS wild-type colorectal cancer

Sang-A Kim^1,*^, Hyejoo Park^2,*^, Kui-Jin Kim^2,†^, Ji-Won Kim^1,†^, Ji Hea Sung^1^, Milang Nam^1^, Ju Hyun Lee^1^, Eun Hee Jung^1^, Koung Jin Suh^1^, Ji Yun Lee^1^, Se Hyun Kim^1^, Jeong-Ok Lee^1^, Jin Won Kim^1^, Yu Jung Kim^1^, Jee Hyun Kim^1^, Soo-Mee Bang^1^, Jong Seok Lee^1^, and Keun-Wook Lee^1^

^1^Division of Hematology and Medical Oncology, Department of Internal Medicine, Seoul National University Bundang Hospital, Seoul National University College of Medicine, Seongnam, 13620, Republic of Korea.

^2^Biomedical Research Institute, Seoul National University Bundang Hospital, Seongnam, 13620, Republic of Korea.

^*^These authors contributed equally to this work.

**^†^Correspondence to:**

Ji-Won Kim, M.D., Ph.D.

Division of Hematology and Medical Oncology, Department of Internal Medicine, Seoul National University Bundang Hospital, Seoul National University College of Medicine, Seongnam, 13620, Republic of Korea.

Tel: +82-31-787-7037

Fax: +82-31-787-4098

E-mail: jiwonkim@snubh.org

Kui-Jin Kim, Ph.D.

Biomedical Research Institute, Seoul National University Bundang Hospital, Seongnam, 13620, Republic of Korea.

Tel: +82-31-787-8406

Fax: +82-31-787-4098

E-mail: kjkim@snubh.org

**Supplementary Figure 1.** Uncropped Western blot images of indicated proteins in whole cell lysates from Caco-2 and SNU-C4 cells treated with AREG and/or cetuximab in Figure 3B.

**Supplementary Figure 2.** Uncropped Western blot images of indicated proteins in whole cell lysates from Caco-2 P, R1, R2, SNU-C4 P, R1, and R2 cells treated with AREG in Figure 5C.

**Supplementary Figure 3.** Uncropped Western blot images of indicated proteins in whole cell lysates from Caco-2 R1 and SNU-C4 R2 cells treated with AREG and/or cetuximab in Figure 5D.
